# Modulation of Molecular Structure and Mechanical Properties of κ-Carrageenan-Gelatin Hydrogel with Multi-Walled Carbon Nanotubes

**DOI:** 10.3390/polym14122346

**Published:** 2022-06-09

**Authors:** Aidar T. Gubaidullin, Anastasiya O. Makarova, Svetlana R. Derkach, Nicolai G. Voron’ko, Aidar I. Kadyirov, Sufia A. Ziganshina, Vadim V. Salnikov, Olga S. Zueva, Yuri F. Zuev

**Affiliations:** 1 Arbuzov Institute of Organic and Physical Chemistry, FRC Kazan Scientific Center of RAS, Arbuzov Street 8, 420088 Kazan, Russia; 2 Kazan Institute of Biochemistry and Biophysics, FRC Kazan Scientific Center of RAS, Lobachevsky Street 2/31, 420111 Kazan, Russia; tat355@mail.ru (A.O.M.); vadim.salnikov.56@mail.ru (V.V.S.); 3 Alexander Butlerov Chemical Institute, Kazan Federal University, Kremlevskaya Street 18, 420008 Kazan, Russia; 4 Department of Chemistry, Murmansk State Technical University, Sportivnaya Street 13, 183010 Murmansk, Russia; derkachsr@mstu.edu.ru (S.R.D.); voronkonikolay@mail.ru (N.G.V.); 5 Institute of Power Engineering and Advanced Technologies, FRC Kazan Scientific Center of RAS, Lobachevsky Street 2/31, 420111 Kazan, Russia; aidarik@rambler.ru; 6 Zavoisky Physical-Technical Institute, FRC Kazan Scientific Center of RAS, Sibirsky Tract 10/7, 420029 Kazan, Russia; sufia@knc.ru; 7 Department of Physics, Kazan State Power Engineering University, Krasnoselskaya Street 51, 420066 Kazan, Russia; ostefzueva@mail.ru

**Keywords:** κ-carrageenan, gelatin, protein-polysaccharide hydrogels, carbon nanotubes, structure, rheological properties

## Abstract

Hydrogels, three-dimensional hydrophilic water-insoluble polymer networks having mechanical properties inherent for solids, have attracted continuous research attention over a long time period. Here, we studied the structure and properties of hydrogel based on gelatin, κ-carrageenan and CNTs using the combination of SAXS, PXRD, AFM microscopy, SEM and rheology methods. We have shown that the integration of polysaccharide and protein in the composite hydrogel leads to suppression of their individual structural features and homogenization of two macromolecular components into a single structural formation. According to obtained SAXS results, we observed the supramolecular complex, which includes both polysaccharide and protein components associated with each other. It was determined that hydrogel structure formed in the initial solution state (dispersion) retains hydrogel supramolecular structure under its cooling up to gel state. The sizes of dense cores of these polyelectrolyte complexes (PEC) slightly decrease in the gel state in comparison with PEC water dispersion. The introduction of CNTs to hydrogel does not principally change the type of supramolecular structure and common structural tendencies observed for dispersion and gel states of the system. It was shown that carbon nanotubes embedded in hydrogel act as the supplementary template for formation of the three-dimensional net, giving additional mechanical strengthening to the studied system.

## 1. Introduction

New “smart” materials such as biomolecular hydrogels are attracting more and more attention due to their enormous potential in various fields of biotechnology and biomedicine [1,2,3,4].

Hydrogels are three-dimensional hydrophilic water-insoluble polymer networks that have mechanical properties inherent for solids, capable of absorbing and retaining a large amount of water or physiological fluid in their structure [5]. More technologically advanced are the “physical” hydrogels in which the spatial polymer network exists due to mechanical entanglement of polymer molecules and/or their intermolecular interactions, including ionic bridges, hydrogen bonding and hydrophobic forces [6,7]. Hydrogels represent a promising system for a wide range of alimentary biomedical applications due to their biocompatibility, excellent permeability for transport of nutrients, metabolites and similarity to the native extracellular matrix [2,3].

In recent decades, hydrogels have received particular attention for complex surgical procedures; they have been used in tissue engineering, dressing and wound healing and to treat injuries or diseases [8]. The development of complex systems for the regeneration of various tissues and organs, such as bones, cartilage and intervertebral discs, presents a major challenge. The designed hydrogel must meet certain requirements for its biomedical use, which include definite fluidity under moderate pressure for use in injections, quick coagulation at the target site, and maintenance of sufficient integrity and mechanical strength [9]. Hydrogels based on natural biopolymers such as polysaccharides and proteins can meet these requirements due to their low immunogenicity, high biocompatibility and susceptibility to degradation by human enzymes [8,10].

Among natural biopolymers, the non-toxic and biodegradable polysaccharide κ-carrageenan is the universal candidate for the food industry [11], pharmacy and tissue engineering [12,13]. κ-carrageenan is a linear sulfated hydrophilic galactan composed of repeating monomer disaccharide units of 1.4-linked α-D-galactose and 1.3-linked β-D-galactose with a variable proportion of sulfate groups at different positions [14]. Formation of water-insoluble hydrogel occurs by water-soluble κ-carrageenan via the polymer chains’ physical entanglement with the ordered structure formation in the junction zones. The gelation of κ-carrageenan is temperature-dependent, with hydrogel forming at low temperatures and melting above a certain temperature upon heating. At high temperatures, the κ-carrageenan polymer chains are randomly coiled; they undergo structural association upon cooling. Various models of percolated polymer networks were proposed for κ-carrageenan [15], including the double helical mode [16,17] and single helix aggregation [18].

The admixture of polysaccharides with proteins is one of the engineering modes for adjustment of gel structure, their gelling and physical-chemical properties [19,20]. In the present study, we used gelatin as the protein component to study the structure and properties of polysaccharide-protein hydrogels. Gelatin is a peptide product of denatured and partially hydrolyzed collagen commonly derived from the animal body by means of its hydrolysis [21]. Gelatin is able to form thermo-reversible hydrogels through the coil-to-helix conformational transition [19]. In carrageenan/gelatin hydrogels, the synergistic effects are determined [22], for example, the increase of mechanical strength [23], flexibility, porosity and water retention capacity [24].

However, despite the high manufacturability and manageable properties of hydrogels, these materials often require additional modifications. Another way to improve physicochemical and mechanical properties of hydrogels is the use of various functional nanomaterials. For biopolymer materials, one of the most promising modifiers is carbon nanotubes (CNTs) [25,26,27]. Biopolymer composites reinforced with carbon nanotubes have potential as artificial conducting media in various fields of biomedical engineering [28], for sustainable energy storage [25,29] and other technological fields which demand electric signaling and modulation of tissue elastic properties.

In this work, the hydrogels consisting of κ-carrageenan, gelatin and multi-walled carbon nanotubes (CNTs) were studied. Previously, we presented the study of a similar system carried out at one, room temperature [30]. The objective of the present study was to examine the influence of CNTs on the κ-carrageenan-gelatin system above and below gelling temperature, namely to compare the influence of multi-wall carbon nanotubes on the structure of the polysaccharide-protein system in the dispersion and gel states. The structural features of these systems were analyzed using a complex of techniques including small-angle X-ray scattering (SAXS), powder X-ray diffraction (PXRD), scanning electron (SEM) and atomic force (AFM) microscopy. The existence of correlation between the structure of the κ-carrageenan-gelatin system and its mechanical properties was demonstrated by rheology studies.

## 2. Materials and Methods

### 2.1. Materials

Gelatin type-A from porcine skin (300 Bloom, G2500, produced by Sigma-Aldrich, St. Louis, MO, USA) with a viscosity-average molecular weight Mη = 100 kDa was used as the protein component of the hydrogel. The isoelectric point (pI) of the gelatin sample was 7–9. Samples of κ-carrageenan produced by Sigma-Aldrich had a viscosity-average molecular weight Mη = 430 kDa and were used without additional purification. The K^+^, Ca^2+^ and Na^+^ ion contents in the polysaccharide sample were below 6, 1 and 1 wt%, correspondingly.

Multi-walled carbon nanotubes (CNTs) from the Taunit carbon nanomaterial (NanoTekhCentr, Tambov, http://www.nanotc.ru/ accessed on 15 May 2022) with an average outer diameter 20–50 nm and the length not exceeding 1000 nm were used as the hydrogel-reinforcing modifier.

To prepare all systems, water purified with the “Arium mini” Ultrapure water system (“Sartorius”, Gottingen, Germany) was used.

### 2.2. Preparation of Solutions, Dispersions and Gels

The following systems were studied: κ-carrageenan-gelatin aqueous dispersion (at 35 °C) and gel (at 14 °C) as well as the CNT-modified versions of these systems.

For every experiment, the fresh samples were prepared in the following order. Aqueous solutions of κ-carrageenan and gelatin were prepared separately. Initially, both biopolymers were swelled in distilled water at 20 °C for 15 h. Then, biopolymers were dissolved with stirring at 70 °C for κ-carrageenan and 50 °C for gelatin. This protocol allowed one to obtain homogeneous colloidal solutions (sols) of polysaccharide and protein. In κ-carrageenan solution, the pH was 8.2, in gelatin solution, pH was equal to 5.2. The initial solutions of biopolymers were mixed with stirring in an ultrasonic bath for 60 min at 50 °C to obtain an aqueous mixture with the desired ratio of κ-carrageenan/gelatin Z = 0.8 (*w*/*w*). The pH value of the mixture was close to 5.5. All studies were carried out in the system containing gelatin in concentration 1.0 wt.% and κ-carrageenan of 0.8 wt.%. At the given pH and biopolymers ratio, neither segregative (thermodynamic incompatibility) nor associative (complex coacervation) macrophase separation [31] was observed. The dispersions were studied at 35 °C, obtained after decrease of temperature from 40 °C. Before measurements, samples were stored at 35 °C for 1 h. To study κ-carrageenan-gelatin hydrogel in the gel state (the gelling temperature of the studied composition is about 20 °C [19,32]), the temperature of the mixture was decreased from 40 to 14 °C. Before the measurements of the system in the gel state, it was stored at 14 °C for 1 h.

To prepare the systems containing carbon material, the CNTs were dispersed in initial gelatin solution using a Bandelin SONOREX TK52 ultrasonic bath (Bandelin, Berlin, Germany, 100 W, 35 kHz) for 60 min [33]. The CNT concentration in all experiments was 3 mg/mL. After centrifugation with microcentrifuge ELMI CM-50M (ELMI, Newbury Park, CA, USA) for 10 min at 10,000 g, the supernatant was taken for preparation of composite hydrogels. The analysis of gelatin ^1^H NMR spectra obtained with Bruker AVANCE III NMR spectrometer (Bruker, Billerica, MA, USA) (600.13 MHz) has shown only minor (~10%) decrease of gelatin content in the supernatant after centrifugation.

### 2.3. Small-Angle X-ray Scattering

Small-angle X-ray scattering (SAXS) was performed with the Nanostar diffractometer (Bruker AXS, Billerica, MA, USA) using CuKα radiation (λ = 1.5418 Å) from a 2.2 kW X-ray tube (40 kV, 35 mA) coupled with Gobel mirrors optics and a HiStar 2D area detector. The beam was collimated using three pinholes with apertures of 1000, 400 and 700 μm. The instrument was operated with a sample-to-detector distance of 63.5 cm to provide data at angles 0.1° < 2θ < 4.8°, which correspond to 0.007 Å−1 < *q* < 0.34 Å−1. The value of *q* is proportional to the inverse length scale (*q* = (4π/λ) sin(θ) Å−1). The measurements were performed in the transition mode using glass capillaries (Capillary Tube Supplies Ltd., Withiel, UK) filled at 40 °C by liquid samples. The capillaries (2 mm diameter) were sealed and put into an evacuated chamber by means of holders. The experiments were carried out at temperatures 14 °C (gel) and 35 °C (sol). For each sample, several experiments were performed, allowing one to control the experiment quality. The acquisition time of diffraction patterns was 10,000 s and 1000 s for determining the absorption coefficient of samples. The data were corrected for the background scattering and absorption of samples. The scattering background (corresponding to a capillary filled with ultrapure water from the “Arium mini” system) was subtracted from all samples. The 2D scattering patterns were integrated using the SAXS program package [34]. Calculation of structural parameters, simulation and graphical representation of results were performed using the SASView [35] and PRIMUS [36] program packages.

To analyze the obtained experimental data, various structural models were used and compared. It was found, that the studied hydrogels can be properly described using the Gauss–Lorentz gel model [37]. This model describes well the scattering from the physical network, which is conventional for the κ-carrageenan-gelatin system in gel and sol states. The SAXS response was modeled as the sum of exponential decay at low *q* (which gives a functional form similar to Guinier scattering) and the Lorentzian at higher *q* [38,39].

### 2.4. X-ray Powder Diffraction

The powder X-ray diffraction (PXRD) measurements were performed on the automatic Bruker D8 Advance diffractometer, equipped with the Vario attachment and Vantec linear PSD using Cu K_α1_ radiation (40 kV, 40 mA), monochromated by a curved Johansson monochromator (λ = 1.5406 Å). Data were collected in the reflection mode with flat-plate samples. Samples were placed on the surface of the standard silicon plate with zero diffraction, which reduces the background scattering. The samples were kept spinning (15 rpm) throughout the data collection. Patterns were recorded in the 2θ range between 3° and 90° in 0.008° steps with a step time of 0.1–4.0 s. Several diffraction patterns in various experimental modes were collected for the samples. Data processing was performed using the EVA and TOPAS software packages [40,41].

### 2.5. Atomic Force Microscopy (AFM)

The study of surface morphology was recorded using an atomic force microscope, Titanium (NT-MDT, Zelenograd, Russia). The measurements were carried out in the open air in the semi-contact mode. Standard silicon cantilevers NSG-10 (NT-MDT, Moscow, Russia) with a force constant of 3.1–37.6 Nm^–1^ and a resonant frequency of 140–390 kHz were used. The Nova PX software was used to operate the microscope. The 2 μL of hydrogel under study was placed on a freshly cleaved mica surface and dried under ambient conditions. All AFM images were obtained at room temperature. The images were processed and analyzed using the Image Analysis program (NT-MDT, Moscow, Russia).

### 2.6. Scanning Electron Microscopy (SEM)

To obtain images by scanning electron microscopy, the pure κ-carrageenan-gelatin mixtures as well as the samples containing CNTs were fixed with 2% glutaraldehyde for 1 h and dehydrated in increasing concentrations of ethanol. Then, the samples were immersed for 3 min in 100% hexamethyldisilazane and the excess of hexamethyldisilazane was removed by filter paper. After this, the samples were transferred to the desiccator for 25 min. After drying, the samples were mounted on special posts and sprayed with platinum. The samples were examined using the Merlin field emission scanning electron microscope (Carl Zeiss, Jena, Germany).

### 2.7. Rheological Measurements

The rheological properties of gels and sols were studied at shear deformation by the MCR 102 (Anton Paar, Graz, Austria) rheometer with a “plate-plate” measuring system (the diameters of upper and lower plates were 50 mm, the gap between planes was 0.5 mm). The sample volume in the cell was 2 mL. The sample temperature control was carried out with the lower heating system and the active casing, both using the Peltier elements P-PTD200 (temperature maintenance error 0.01 K). The variation of the given temperature was within ±0.1 °C. Measurements were carried out in the following deformation modes: periodic oscillations at constant temperature with varying amplitude, ω, at constant frequency, ω = 6.28 s^−1^, or varying frequency, ω, at constant amplitude, γ = 1%, the range of γ was 0.9–139% and ω was 0.0671–23.8 s^−1^. The experiments were performed at temperatures 14 °C and 35 °C. In the first case (T = 14 °C), after loading of the sample into the measuring system, it was kept for 60 min at ω = 0.2 s^−1^ and γ = 0.2% until the stability of data (elastic modulus) was achieved, which means the gelation of the sample. Further deviation of the elastic modulus did not exceed 2%. For the second case, the sample was held at T = 35 °C for 5 min before measurement since the longer exposure led to its intense drying.

## 3. Results

### 3.1. PXRD Overview of Hydrogel Phase State

**Figure 1:**
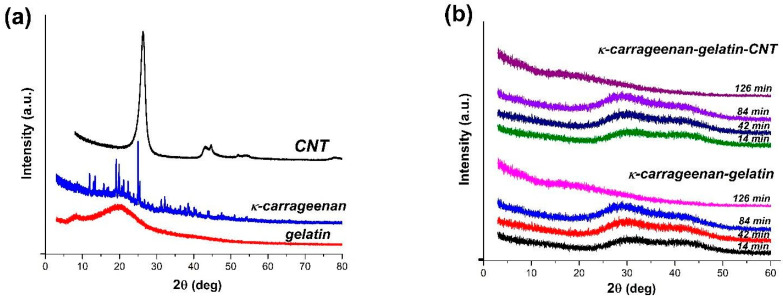
Diffraction patterns from dry original components (**a**) of hydrogel and time evolution of X-ray diffraction of κ-carrageenan-gelatin hydrogels upon drying with and without CNTs (**b**).

According to the powder X-ray diffraction data (PXRD), two original components, CNTs and gelatin, are the weakly ordered systems; the third one, κ-carrageenan, forms a stable crystalline phase (Figure 1a). The κ-carrageenan-gelatin hydrogel as well as hydrogel with addition of CNTs were studied by the PXRD method during their natural drying on the surface of a silicon plate. Both samples in the gel state are characterized by the same type of diffraction pattern in the form of two strongly broadened amorphous halos (Figure 1b), corresponding to the average interatomic distances. In the process of sample drying, the peaks degenerate into one small broadened peak in the interval of diffraction angles 10–15 degrees. Its low intensity is associated with a small amount of scattering substance obtained after gel drying. The addition of nanotubes to hydrogel does not change the original diffraction pattern of the generally amorphous hydrogel sample.

The integration of polysaccharide and protein in the composite hydrogel leads to suppression of their individual structure, which is depicted in Figure 1b as the diffraction pattern is typical for amorphous substances with short-range order. This fact indicates indirectly the homogenization of the system without separation of initial components into individual subsystems. The addition of nanotubes results in an even more stabilizing effect on the hydrogel. Despite the presence of nanotubes, which are clearly observed in all samples by the naked eye, their presence is not detected in the diffraction patterns of the hydrogel.

The phase homogeneity of the studied systems, confirmed by the PXRD method, does not exclude the processes of aggregation and segregation in samples at all scale levels, which, in turn, can be estimated in a hierarchical order by small-angle X-ray scattering, atomic force and scanning electron microscopy.

### 3.2. SAXS Structural Characterization of Dispersions and Gels

**Figure 2:**
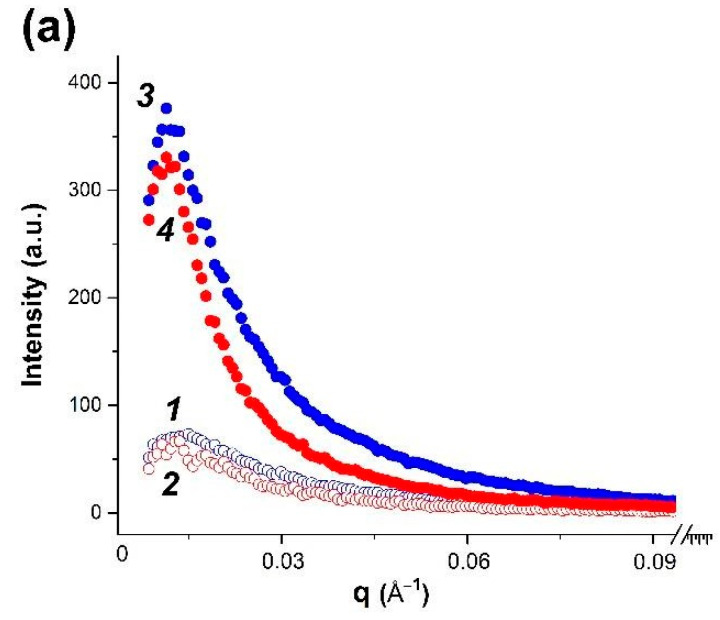

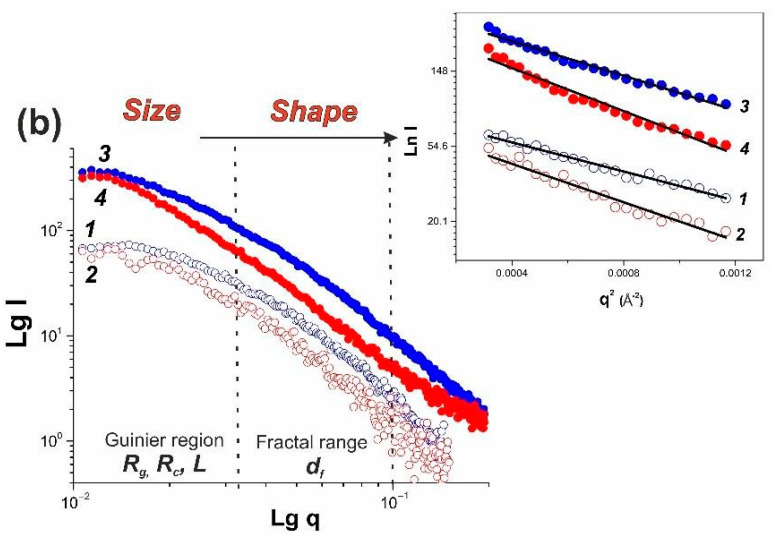
Experimental SAXS curves for pure (open symbols) and modified by CNT (filled symbols) κ-carrageenan-gelatin systems (1, 3—14 °C; 2, 4—35 °C) in *I*(*q*) scale (**a**) and double logarithmic scale (**b**) after background substruction. Scattering vector *q* = 4πSinθ/λ, Å^−1^. (**b**) marks the characteristics which can be extracted from different angular regions. Insert shows linear approximation of Guinier plot.

After integration of two-dimensional experimental SAXS patterns, the one-dimensional curves of small-angle scattering were obtained (Figure 2), with the shape typical for systems of non-interacting particles. A rather high scattering intensity indicates structural microheterogeneity of systems with the presence of randomly oriented scattering particles (zones of increased density), corresponding in their size to the range of SAXS methodology (1–100 nm) [42]. High intensity of SAXS scattering in κ-carrageenan-gelatin hydrogels is associated apparently with participation of both components in microphase formation. The presence of carbon nanotubes results in even greater scattering intensity in comparison with pure hydrogel. In order to analyze the morphology of hydrogels studied, a number of structural characteristics were calculated basing on the SAXS experimental data.

One of the important non-structural characteristics obtained from the power law dependences of intensity and scattering angle is the particle gyration radius *R_g_* [40]. For the studied systems, this parameter was calculated by two independent methods, based on the analysis of diffraction data in direct and reciprocal spaces [42,43]. On the basis of the obtained radius of gyration, it is possible to analyze the size of particles or heterogenic domains. Thus, assuming a spherical shape of particles, one can calculate from *R_g_* the effective average particle radius *R_sph_* (Table 1) using the expression (*R_sph_* = *R_g_*$\sqrt{5/3}$) [42].

Earlier, based on the FTIR spectroscopy and molecular docking results, we proposed [19] that in the studied hydrogel, the most energetically favorable configuration of junction is formed by gelatin triple helix segments and κ-carrageenan double helix. Both initial components of composite hydrogel and their junctions are rod-shaped and rigid [19], resulting in the anisotropic structural network, which can be hypothetically considered to be elongated particles between nodes. Such a highly anisotropic system can be modelled as the ensemble of cylindrical particles with average length *L* and cross-section *r_c_*, obtained using the modified Guinier plots (ln(q*I(*q*)) vs. *q*^2^) [43]. These parameters are also presented in Table 1. Our data show that addition of CNT does not change the type of obtained supramolecular structure, increasing only particle size with increase of temperature. An analysis of particle gyration radii both in spherical and anisometric approximations indicates that in the presence of CNT the sample morphology is similar to that of the initial gel.

**Figure 3:**
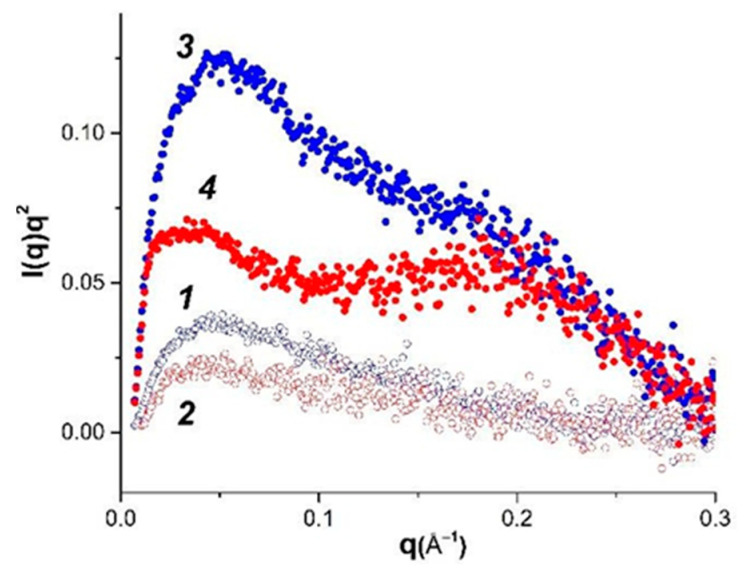
Kratky plot for pure (open symbols) and modified by CNT (filled symbols) hydrogel κ-carrageenan-gelatin systems (1, 3—14 °C; 2, 4—35 °C).

Fractal particle dimensions d_f_, obtained from SAXS data (Figure 2), characterize the “smoothness” of “particle” surface. The fractal conception is introduced for objects of complex configuration, which cannot be measured in standard length scale. The Kratky plots for hydrogel at 14 °C and aqueous dispersion at 35 °C (Figure 3) indicate that formation of mass fractals, which related the mass M of the fractal object to its radius, is typical for these samples. The slope of the log−log plot of scattering intensity vs. scattering vector *q* shows in its medium range a power law behavior (Porod exponent) for all samples. The d_f_ values obtained from the SAXS curve slope are presented in Table 2. It is generally accepted [44,45] that the d_f_ value close to 1 reflects the linear structures, the values about 2 characterize the smooth surfaces of semi-swollen coils, and a further increase of fractal dimension to 3 is observed for more strongly branched networks. The obtained d_f_ values (Table 2) correspond to the physically crosslinked gels exhibiting scattering behavior close to polymer coils swollen in a good solvent [46]. The presence of CNTs slightly stabilizes the hydrogel network.

The shape of SAXS, plotted as the Kratky plot curves in the range of large wave vector values (Figure 3), characterizes the hydrogel supramolecular structure. An analysis of these graphs makes it possible to distinguish the scattering from the rod-shaped supramolecular structures, Gaussian chains and bulk mass fractal structures. For example, the linear shape of the scattering intensity dependence at large *q* vector values on the Kratky plots characterizes the scattering from the rod-like structures. The Kratky plots with intensity, monotonically increasing with *q* and reaching a plateau value at high *q*, indicate the scattering of Gaussian chains. The scattering from a fractal three-dimensional structure shows a distinct peak on the Kratky plots. In this study, the analysis of the Kratky graphs shows that hydrogels can be alternatively analyzed as the system of flexible cylindrical particles with a cross-section much smaller than their length in the dispersion state (35 °C), but in the gel state (14 °C), the volumetric three-dimensional structures of globular type are more probable.

**Figure 4:**
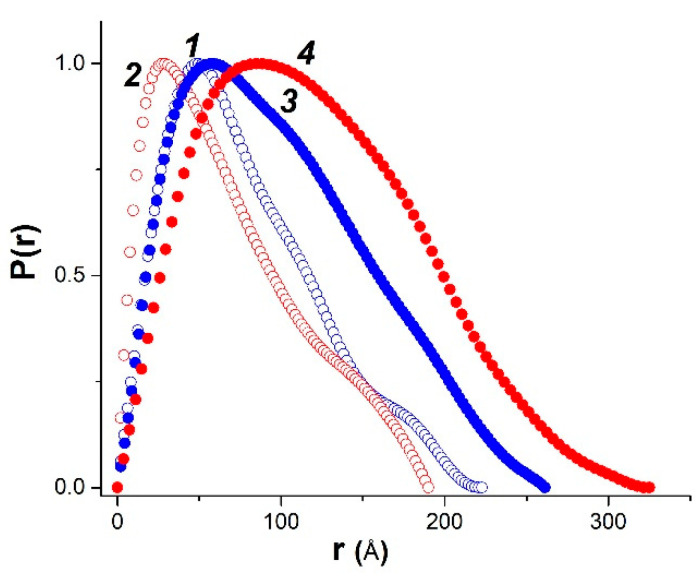
Calculated normalized distance distribution functions *P*(*r*) derived from SAXS for pure (open symbols) and modified by CNT (filled symbols) κ-carrageenan-gelatin systems in hydrogel and aqueous dispersion states (1, 3—14 °C; 2, 4—35 °C).

In addition, we used the SasView [35] and PRIMUS programs [35] for modeling and fitting the experimental SAXS scattering curves in the framework of various structural model representations of studied samples. The comparison of experimental scattering curves in the framework of various structural models, as well as their comparison with literature data, made it possible to identify several options which give the most effective description of experimental data with adequate and clear physical meaning. In particular, the modeling of scattering curves by the PRIMUS program within the framework of the globular model with the local monodisperse environment makes it possible to determine the distance distribution function *P*(*r*), which yields a real-spaced size of particles (Figure 4). The *P*(*r*) value equals to zero if r exceeds the maximum characteristic size in particle (*D_max_*), and this allows one to estimate *D_max_* from the experimental data using the so-called indirect transform methods [47]. The shape of *P*(*r*) functions provides the information on the overall particle shape and gives the independent method for *R_g_* determination.

The obtained *R_g_* and *R_c_* values for studied samples (Table 1) were used in the modelling of the hydrogel structure using standard structural models, included in the SAXSView software packages. These models are based on the combination of known regularities in the SAXS intensity distribution curves, for example, as it is presented in the Beaucage model [48], and make it possible to obtain the same values of *R_sph_* and *R_c_*, as well as a number of other parameters. We analyzed a significant number of models presented in these programs to choose an adequate physical model for our system. An analysis of literature data on the gel structure, including those based on gelatin and κ-carrageenan [49,50], indicates that the most commonly used models are the model of cylindrical particles in gels [51,52] and the Gauss–Lorentz (G-L) gel model [37,53]. The dimensional characteristics obtained within the Gauss–Lorentz (G-L) gel model are summarized in Table 3. The results of the experimental scattering curves modelling are shown in Figure S1 (see Supplementary Information).

In the Gauss–Lorentz gel model, within one temperature, the change in the dynamic correlation length, associated with the physical network of entanglements, turns out to be insignificant when going from pure systems to the CNT-modified ones, while the static correlation length noticeably increases, indicating an increase in the size of the molecular coils in the modified systems.

### 3.3. AFM Study of Hydrogel Morphology

**Figure 5:**
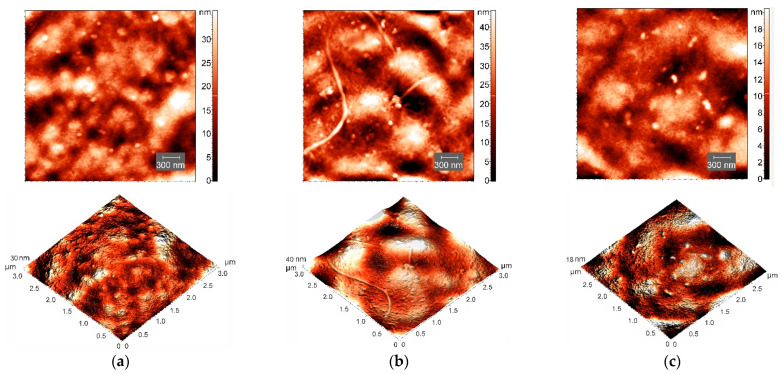
AFM images of xerogel (dried hydrogel) films and corresponding 3D surface visualization of samples of pure κ-carrageenan-gelatin hydrogel (**a**) and this hydrogel with CNTs (**c**). Film from 0.8 wt.% κ-carrageenan solution (**b**) is shown for comparison.

AFM experiments [54] provided direct structural visualization of polysaccharide-protein hydrogels at the nanoscale and illustrated the behavior of the studied system in the presence of CNT. Figure 5a,c show the AFM surface images of xerogel (dried hydrogel) films without and with CNTs, correspondingly. Upon drying of κ-carrageenan-gelatin hydrogel, which is necessary for the AFM experiment, one can see (Figure 5a) the formation of the biopolymer network, the so-called biopolymer scaffold [55]. In this composite hydrogel, the fibers responsible for gel formation are shorter compared with those of pure κ-carrageenan solution (Figure 5b) and have a thickness of about 30 nm. The introduction of CNTs into κ-carrageenan-gelatin hydrogel did not principally affect the surface morphology of the film surface (Figure 5). The AFM image (Figure 5c) also contains a network structure and small agglomerates 70–100 nm in size, similar to those in κ-carrageenan and pure composite gel (Figure 5a). The AFM data confirm that nanotubes are uniformly distributed, being packed/wrapped with biopolymers.

To characterize the surface topography of AFM images, the root-mean-square (*RMS*) parameter was analyzed. The *RMS* (*R_q_*) roughness parameter is a measure of departures of profile from the mean level, which is equivalent to standard deviation of heights of peaks [56]. The addition of CNTs to κ-carrageenan-gelatin system results in the lowering of surface roughness to *R_q_* = 4.13 nm compared with *R_q_* = 6.98 nm in the CNT-free κ-carrageenan-gelatin xerogels, which is consistent with the known results [57]. The decrease in the roughness value upon the CNTs’ addition may indirectly indicate “stabilization” of the supramacromolecular structure of xerogel and sufficiently uniform distribution of CNTs in the κ-carrageenan-gelatin matrix. This finding qualitatively confirms the results of fractal analysis coming from SAXS data.

### 3.4. SEM Visualization of Hydrogel Morphology

**Figure 6:**
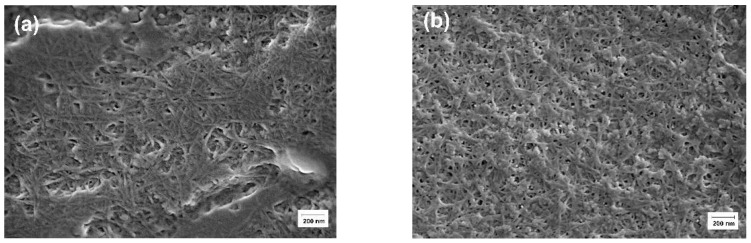
SEM images of κ-carrageenan-gelatin hydrogels: pure (**a**) and with CNTs (**b**).

The morphology of κ-carrageenan-gelatin hydrogels was characterized by scanning electron microscopy. The SEM images show that the samples are in a homogeneous state with a smooth surface (Figure 6), which is consistent with the X-ray powder diffraction data (Figure 1). The images show good dispersity of CNTs in the hydrogel matrix without visible agglomeration. In addition, one can see a more regular polymer network with slightly thinner polymer bundles in the presence of CNTs (Figure 6b) in comparison with pure κ-carrageenan-gelatin hydrogel (Figure 6a).

### 3.5. Rheological Characterization of Pure and CNT-Modified Hydrogels

**Figure 7:**
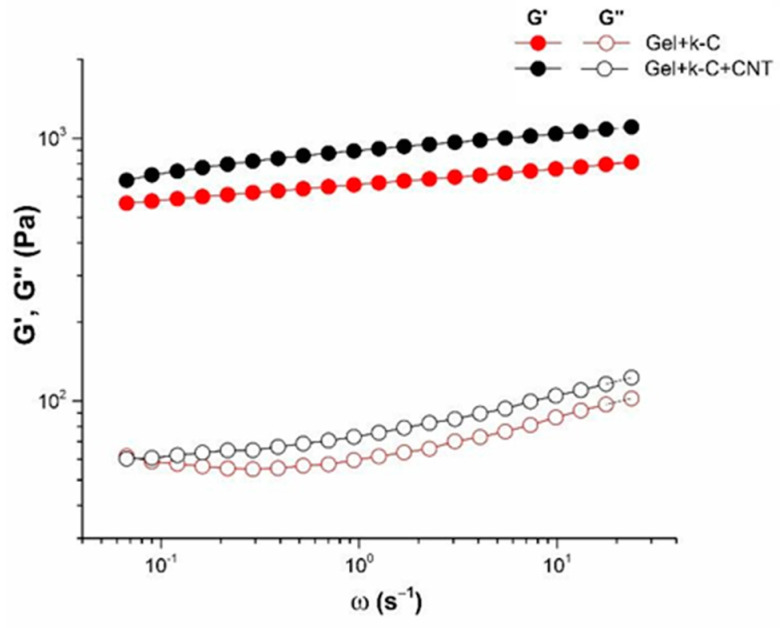
Frequency dependencies of storage (closed symbols) and loss (open symbols) moduli for pure (red) and CNT-modified (black) hydrogels at 14 °C, γ = 1%.

Viscoelastic properties of the κ-carrageenan-gelatin and κ-carrageenan-gelatin-CNT hydrogels are determined by the structure of gel as a solid-like body. In the region of linear viscoelasticity, hydrogels demonstrate independence of the storage modulus from frequency (Figure 7), which indicates the solid-like behavior of material with a certain structural rigidity and strength. In this case, the storage modulus significantly exceeds the loss modulus G′ > G″, demonstrating the dominance of elastic nature over the viscous one. The measured rheological properties make it possible to characterize the studied hydrogels as physical gels. It is fully applied to both the pure hydrogel and that modified with CNT. So, experimental data show that nanotube additivity affects the gel’s elastic characteristics, increasing the storage modulus. Obviously, this is a consequence of formation of a more “rigid” structure in the CNT-modified system [58,59].

**Figure 8:**
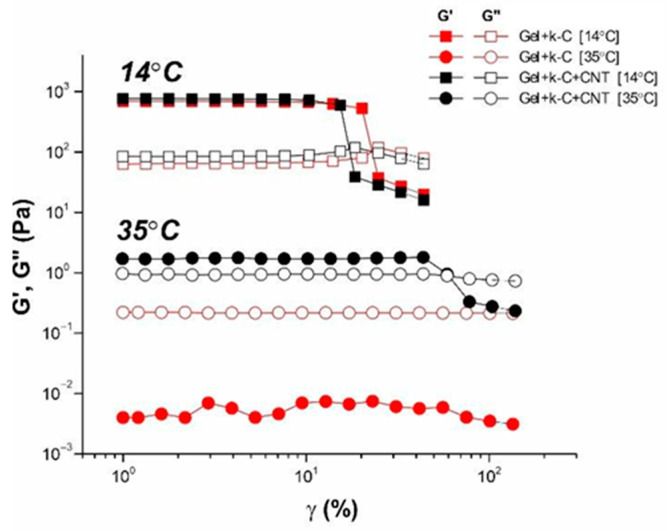
Amplitude dependencies of storage (closed symbols) and loss moduli (open symbols) at ω = 6.28 s^−1^ for pure (red) and CNT-modified (black) gel at 14 °C (squares) and dispersion at 35 °C (circles).

The linear and non-linear behavior of the studied systems in the gel and dispersion states was studied by the method of large amplitude oscillatory shear (strain sweep measurements). Figure 8 depicts the dependences of the storage and loss modulus on the amplitude for polysaccharide-gelatin gel and dispersion without and with CNT addition. At low amplitudes, the elastic modulus does not change, showing the linearity of mechanical behavior. An increase in the strain amplitude above some critical value leads to a sharp drop in the elastic modulus, which indicates a transition to the non-linear regime of mechanical properties. The polysaccharide-gelatin hydrogels retain the linearity of mechanical properties up to app. 20% of strain amplitude, demonstrating a slightly more mechanically robust network compared with hydrogels modified by CNT. The CNT addition slightly reduces the linearity limit of the viscoelasticity region. The gel breaking point, γ*, obtained from the crossover point at which G′ = G′′, indicates a transition from a solid-like to a liquid-like state of matter [60]. This effect is due to the destruction of the three-dimensional gel structure at large strains. The decrease in the value of breaking point for CNT-modified systems (γ* = 14%) compared to the pure ones (γ* = 20%) may indicate the formation of more fragile structural gel networks in the presence of nanotubes. It is interesting to note that for the system in the CNT-modified sol state, storage modulus exceeds loss modulus G′ > G″, indicating the armoring effect of CNT even in polymer dispersion.

## 4. Discussion

All applied methods display the influence of CNTs on the structure and properties of κ-carrageenan-gelatin hydrogel, which we shall discuss in the following section. We start from SAXS data since they give more miscellaneous and deep information on the studied systems. In order to analyze the morphology of studied systems, some structural parameters were calculated from small-angle X-ray scattering data, which are compared with the known structural models.

**Figure 9:**
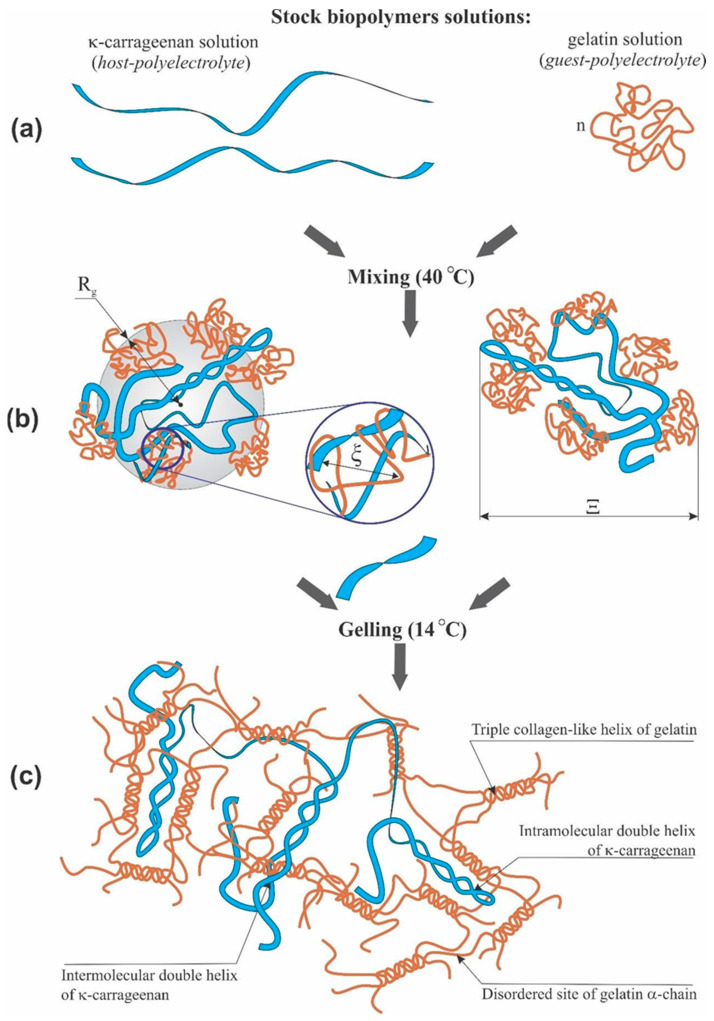
Qualitative scheme of κ-carrageenan and gelatin initial conformations (**a**), basic sizes of κ-carrageenan and gelatin complexation (**b**) and scheme of polysaccharide-protein hydrogel (**c**).

Earlier, some of our co-authors proposed the interaction mechanism and structural details of the κ-carrageenan-gelatin polyelectrolyte complex in aqueous phase (Figure S2, see Supplementary Information) [17,19,54]. Thin atomic organization of this polysaccharide-protein supramolecular complex may be altered depending on the origin of biopolymers and the acid-base conditions of their forming. Methods, applied here, are aimed at the information about the next level of the κ-carrageenan-gelatin gelling system, namely about the size of static inhomogeneities in the studied systems in the dispersion and gel states, including the disturbing influence of CNTs. We applied the SAXS methodology and techniques as the main source of quantitative and numerical information about the subject of research, in accordance with the scheme and dimensional definitions depicted in Figure 9.

The structure of the first system (dispersion) practically determines the structure of the second system (gel). At 35 °C (we mixed two systems at 40 °C and studied them at 35 °C), our system is the water dispersion of two biopolymers. The physical network of macromolecular engagement is the fluctuating one—the nodes of this net can be destroyed as a result of thermal motion and appear in a new place. This process is determined by gelatin conformation, which at this temperature has a conform of statistical coil. In the gel state (14 °C), the three-dimensional structural network of physical gel becomes stronger and determines the properties of the system as a solid body, i.e., mechanical strength of gel and the absence of fluidity. At this temperature, gelatin takes the helix conformation. The gel network is formed as a result of strong binding sites formed by a triple collagen-like helix of gelatin. The knots are strengthened by the interaction of these triple helixes with double helix of κ-carrageenan.

Using the SAXS method, the inhomogeneities of colloidal size (1–100 nm) are studied [55]. At inhomogeneities of such density, we considered the following structural units of κ-carrageenan-gelatin system, shown in Figure 9. According to our knowledge, the main structural element of this system is the polysaccharide-protein polyelectrolyte complex (PEC). Early, it was shown by some of our co-authors [56,57] that at the used κ-carrageenan/gelatin weight ratio (0.8) there are no free protein or polysaccharide molecules, but almost all of them are bound in the polyelectrolyte complexes. Their size and averaged spatial arrangement can be characterized by parameters shown in Figure 9. They are the PEC radius of gyration (*R_g_*) and the PEC mean size Ξ, and ξ is the correlation length of intramolecular interactions between the fluctuating chains of polymer, i.e., some zones of intramolecular hardening [37]. The main distinction of the system in the gel state consists in the presence of connectors (or crosslinking) (Figure 9 in the bottom), combining individual PECs into a spatial 3D network via the complexation of gelatin triple helixes with one chain or double helix of κ-carrageenan molecules [19].

The results of the SAXS data analysis using different theoretical models are presented in Table 1, Table 2 and Table 3. The values of the apparent radius of biopolymer coils for κ-carrageenan and gelatin were estimated at infinite dilution as 390 Å and 90 Å, correspondingly [56]. Data presented in Table 1 show the apparent sizes of the pure hydrogel structural elements, which have the same order of magnitude, both for spherical and cylindrical models. We suppose that there is no principal contradiction between the sizes of individual macromolecular coils from the literature data and the PEC parameters obtained in our experiments. The slightly lesser dimensions obtained in our study are due to substantially dense packing of polymer chains in PECs helping with the huge number of close intermolecular contacts between polysaccharide and protein, determined by hydrophobic interaction, electrostatic ion pairing and hydrogen bonding [54].

Table 1 shows that the PEC gyration radius *R_g_* decreases visibly under the gelling; this can be explained by the formation of additional and stronger junction zones between biopolymers [19]. The formation of additional network junctions between gelatin triple helices and κ-carrageenan double helices is suggested to be the reason for the excess strengthening of κ-carrageenan-gelatin gel in comparison with corresponding dispersion at higher temperatures shown in the rheological experiment. A similar tendency (Table 3) is depicted in the framework of the Gauss–Lorentz gel model for estimation of the SAXS-based static and dynamic correlation lengths.

**Figure 10:**
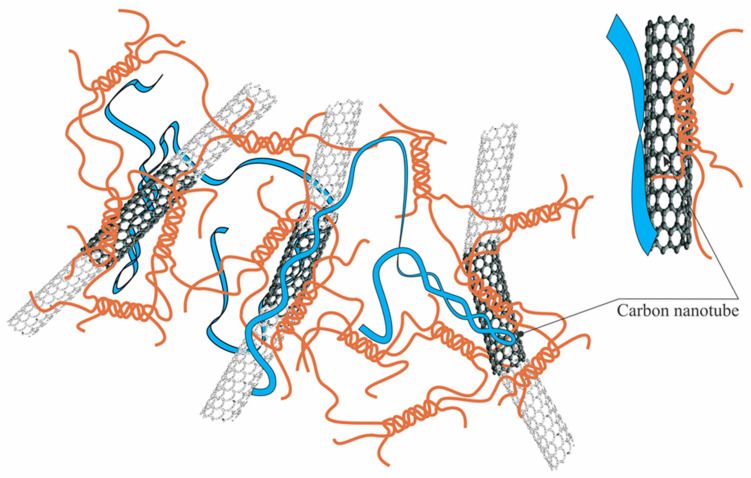
Interaction of κ-carrageenan-gelatin PEC complexes with CNT surface. Figure shows only short fragments of long CNTs.

Analyzing the influence of CNTs on the κ-carrageenan-gelatin hydrogel spatial arrangement, one has in view the size and spatial concentration of carbon nanotubes. According to the manufacturer’s information, the used CNTs have an average outer diameter of 20–50 nm and a length not exceeding 1000 nm. The CNT volume fraction does not exceed 0.2–0.3 volume percent. Thus, in the presence of CNTs, we have a rather loose carbon net. The presence of CNTs results in a slight increase of *R_g_* (Table 1) in gel and dispersion states, arising evidently as a result of CNTs embedding into the supramolecular architecture of the hydrogel (Figure 10). It is remarkable that according to SAXS data the presence of CNTs does not absolutely break the existing hydrogel structure at different size scales.

It should be noted that structural features of CNTs determine hydrophobic properties of their surface. Therefore, polymers containing hydrophobic units (for example, block copolymers) could be effective for CNTs’ dispergation [61]. The interaction of individual fragments of the biopolymer chain, in particular, gelatin segments, with the CNT surface, has a hydrophobic nature. The increase of interactions between polyelectrolyte chains in the presence of CNTs was mentioned in [62]. This phenomenon was explained by the hydrophobic effect of CNTs and the increasing crosslinking density between CNTs and gelatin under the increase of CNTs’ concentration. In addition, in a solution of natural polyelectrolytes, one should not underestimate the occurrence of additional electrostatic interactions due to polarization of CNTs, resulting in the appearance of a bound negative charge on their surface [63] and appearance of a double electric layer at the CNT interface. These phenomena can also lead to an increase in electrostatic interactions, including strong Coulomb and weak Van der Waals ones, of biopolymer chains with the CNT surface.

Thus, it can be argued that transition from the dispersion (35 °C) to the gel (14 °C) state does not lead to cardinal changes in the sample morphology, which, to a certain extent, indicates the stabilization of the system. The carbon nanotubes, present in the system, act as a framework and template for formation of the three-dimensional network. An analysis of the structure of the original and CNT-modified gels within the framework of the Gauss–Lorentz gel model shows good agreement between experimental and calculated SAXS curves. It has been established that, within the same temperature, the change in dynamic correlation length, associated with the physical network of junctions, is insignificant in regards to modification by CNTs. At the same time, the statistical correlation length noticeably increases, which indicates an apparent increase in the size of molecular coils in the modified gel.

Despite the relatively low volume fraction of CNTs, their uniform distribution over the hydrogel bulk leads to their noticeable influence on sample morphology and properties. Besides the direct structural impact of CNTs, detected well by PXRD and SAXS at various supramolecular levels, one can see the weak “global” effects touching upon the all-volume and all-surface morphology. It is clearly seen during the analysis of the AFM experiment (Figure 5), which confirms that nanotubes are distributed uniformly over the polysaccharide-protein system with visible uniform perturbation of its structure. The similar “carpet” action of CNT on the κ-carrageenan-gelatin system is shown in the SEM images (Figure 6). One can see rather homogeneous action of CNTs on the hydrogel matrix, with slightly thinner polymer bundles in the presence of CNTs (Figure 6b) in comparison with the pure polysaccharide-protein system (Figure 6a).

The same tendency of mechanical strengthening of the κ-carrageenan-gelatin hydrogel is shown by rheological study. First, the increase of the system strength is detected by temperature decrease of transition from the fluid dispersion to the solid-like gels. Second, the implantation of CNTs provides additional mechanical properties to the hydrogel, both in the gel and dispersion states. Nevertheless, the CNT addition slightly reduces the linearity limit of the viscoelasticity region, giving more fragile structure.

**Figure 11:**
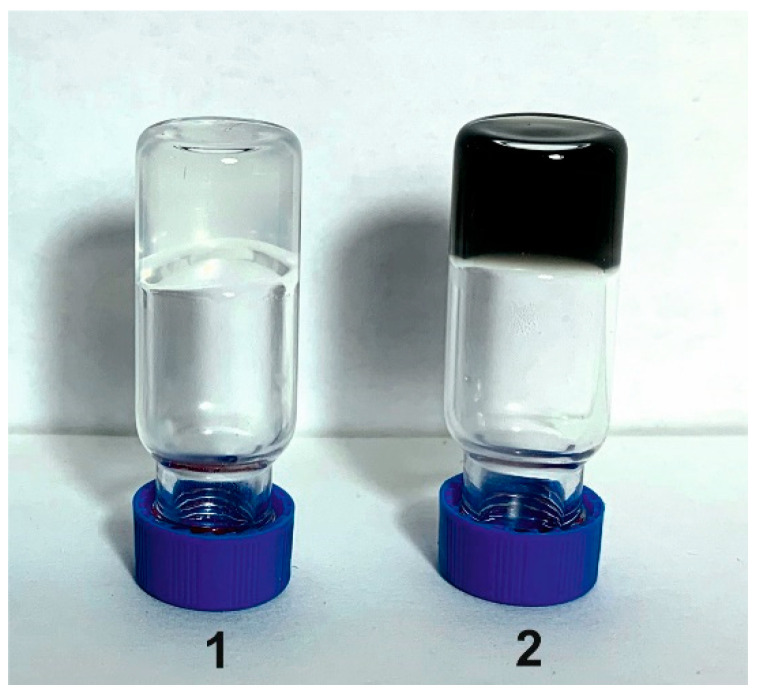
The reinforcing properties of CNTs on κ-carrageenan-gelatin hydrogels at 23 ± 2 °C. 1-without CNTs; 2-with CNTs.

In addition, we have detected the alteration of some physicochemical properties of κ-carrageenan-gelatin hydrogel provoked by dispersed CNTs. In spite of the limited quantity of CNTs capable of being accepted by the composite biopolymer host, the reinforcing properties of carbon nanotubes could be seen quite distinctly. These CNT characteristics are detected not only by our results on the rheological behavior of κ-carrageenan-gelatin hydrogels, but also in the simple tests of the hydrogel gelling (Figure 11).

## 5. Conclusions

The hydrogels based on gelatin, κ-carrageenan and CNTs have been successfully prepared and characterized. The experiments carried out gave a clear picture of the structure and properties of nanocomposite κ-carrageenan-gelatin hydrogels. The results obtained indicate that the combination of SAXS, PXRD, AFM microscopy, SEM and rheology is an effective approach for characterizing the structure and properties of a CNT-reinforced protein-polysaccharide hydrogel. Using the X-ray methods, we have surely shown that the structure formed during preparation of the κ-carrageenan-gelatin hydrogel in the initial (dispersed) state at high temperature determines the supramolecular structure of the system and in the gel state.

The phase homogeneity of the studied systems was confirmed by the PXRD method. According to these data, the samples of composite hydrogels are amorphous and there are no signs of crystallization of individual components, even in the case of modification with CNT. The integration of polysaccharide and protein in the composite hydrogel leads to suppression of their individual structure. We have shown that there is homogenization of two macromolecular components and unification into single structural formations and the presence of CNT introduces a stabilizing effect into this process.

According to our SAXS results, we have observed the supramolecular complex which includes both polysaccharide and protein components which are associated with each other. The sizes of dense cores of these polyelectrolyte complexes (PEC) slightly decrease in the gel state in comparison with PEC water dispersion. Calculation of spherical PEC “particles” radii from the radii of gyration and their comparison with obtained maximum distances of particles indicate their noticeable deviation from the spherical shape. Appropriate calculations within the concept of anisometric (elongated) particles and comparison of these data convince us of this assumption. In this case, an increase in the linear dimensions of such ellipsoidal particles in the dispersed state (35 °C) is quite natural, but such changes are not accompanied by alterations in morphology and the type of formed particles. Thus, in the absence of external influences on this system, the transition of hydrogel from dispersion to the gel state does not lead to cardinal changes in sample morphology, with definite stabilization of the system owing to some compactization of PECs.

The addition of CNTs to the gel does not lead to a fundamental change in the type of supramolecular structure and common tendencies observed for dispersion and gel states of the system, leading only to a certain increase in particle size. Moreover, the analysis of the gyration radii both in the spherical and anisometric approximations indicates a sample morphology similar to that of the initial gel. It can be argued that carbon nanotubes embedded in the system act as a framework or template for the formation of the three-dimensional network. The analysis of the structure of the original and CNT-modified system within the framework of the Gauss–Lorentz gel model shows good agreement between the experimental and calculated small-angle scattering curves. It was established that the change in dynamic correlation length associated with the physical network of links is insignificant in the transition from pure hydrogel to gel modified with CNTs. At the same time, statistical correlation length noticeably increases, indicating the increase in the size of molecular coils in the modified gel.

It has been shown that the incorporation of CNTs into polymer composites significantly improves their mechanical properties. Based on the experiments performed, we proposed the structure of physically cross-linked hydrogels without and with CNT addition. The CNT acts as a multifunctional crosslinking agent for polymer chains. The obtained information on the structure and properties of the resulting hydrogel can promote the development of new materials with improved properties for targeted applications in biomedicine and biotechnology, as well as in any other relevant areas.

## Figures and Tables

**Table 1:** Structural parameters for pure and CNT-modified systems in aqueous dispersion and hydrogel states.

	Structural Parameter, Å	T, °C	Pure System	CNT-Modified System
**Sphere**	***R_g_***	14	56.0 ± 0.3	73.0 ± 1.3
		35	66.2 ± 0.5	81.1 ± 2.2
	$R_{\mathrm{sph}}=R_{g}\sqrt{5/3}$	14	72.3	94.2
		35	85.5	104.7
	***D_max_***	14	222.7	241.3
		35	239.6	324.2
**Cylinder**	***R_c_***	14	21.21 ± 0.07	28.1 ± 0.8
		35	21.34 ± 0.05	46.2 ± 0.9
	$r_{c}=R_{c}\sqrt{5/3}$	14	27.4	36.3
		35	27.6	59.7
	$L=\sqrt{12\cdot \left(R_{g}^{2}-R_{c}^{2}\right)}$	14	179.5	233.5
		35	208.4	230.9

**Table 2:** Fractal dimension d_f_ for pure and CNT-modified systems in aqueous dispersion and hydrogel states.

T, °C	Pure System	CNT-Modified System
14	1.7	2.05
35	1.98	2.11

**Table 3:** Intrinsic dimensions of κ-carrageenan-gelatin aqueous dispersion (at 35 °C) and hydrogels (at 14 °C) for Gauss–Lorentz gel model presentations.

G-L Gel Model	T, °C	Pure System	CNT-Modified System
Ξ (Å) ξ (Å)	14	64.3 ± 0.3	86.8 ± 0.1
		29.17 ± 0.08	29.7 ± 0.2
Ξ (Å) ξ (Å)	35	99.95 ± 0.09	122.0 ± 0.2
		37.9 ± 0.1	44.3 ± 0.8

Ξ—length scale of static correlations in gel; ξ—dynamic correlation length, attributed to vibrations of polymer chains between crosslinks.

## Data Availability

The data in this study are available on reasonable request from the corresponding author.

## Supplementary Materials

The following supporting information can be downloaded at: https://www.mdpi.com/article/10.3390/polym14122346/s1, Figure S1. Fitting of experimental SAXS curves (in logarithmic scale, *lg(I)* vs. *q*) in the Gauss–Lorentz gel model framework for pure hydrogel (a—14 °C; b—35 °C) and hydrogel modified by CNT (c—14 °C; d—35 °C) after background substruction, experimental points—circles, solid line—calculated curves. Scattering vector *q* = 4πSinθ/λ, Å^−1^. Figure S2. Qualitative scheme of κ-carrageenan-gelatin polyelectrolyte complex and its gelling via triple collagen-like helixes and disordered α-gelatin chains bonding.
