# Supplementary material for: Modulation of Molecular Structure and Mechanical Properties of κ-Carrageenan-Gelatin Hydrogel with Multi-Walled Carbon Nanotubes
Source: Polymers (Basel). 2022 Jun 9;14(12):2346. doi: 10.3390/polym14122346 (PMC9229921; doi:10.3390/polym14122346)
Supplement: Supplementary file 1 [file polymers-14-02346-s001.zip › polymers-1751631-supplementary.pdf]

Modulation of Molecular Structure and Mechanical Properties of κ-Carrageenan-Gelatin Hydrogel with Multi-Walled Carbon Nanotubes

Aidar T. Gubaidullin [1,†,*], Anastasiya O. Makarova [2,3,†], Svetlana R. Derkach [4], Nicolai G. Voron′ko [4], Aidar I. Kadyirov [5], Sufia A. Ziganshina [6], Vadim V. Salnikov [2], Olga S. Zueva [7] and Yuri F. Zuev [2,3,*]

[1] Arbuzov Institute of Organic and Physical Chemistry, FRC Kazan Scientific Center of RAS, Arbuzov Street 8, 420088 Kazan, Russia
[2] Kazan Institute of Biochemistry and Biophysics, FRC Kazan Scientific Center of RAS, Lobachevsky Street 2/31, 420111 Kazan, Russia; tat355@mail.ru (A.O.M.); vadim.salnikov.56@mail.ru (V.V.S.)
[3] Alexander Butlerov Chemical Institute, Kazan Federal University, Kremlevskaya Street 18, 420008 Kazan, Russia
[4] Department of Chemistry, Murmansk State Technical University, Sportivnaya Street 13, 183010 Murmansk, Russia; derkachsr@mstu.edu.ru (S.R.D.); voronkonikolay@mail.ru (N.G.V.)
[5] Institute of Power Engineering and Advanced Technologies, FRC Kazan Scientific Center of RAS,
Lobachevsky Street 2/31, 420111 Kazan, Russia; aidarik@rambler.ru
[6] Zavoisky Physical-Technical Institute, FRC Kazan Scientific Center of RAS, Sibirsky Tract 10/7,
420029 Kazan, Russia; sufia@knc.ru
[7] Department of Physics, Kazan State Power Engineering University, Krasnoselskaya Street 51, 420066 Kazan, Russia; ostefzueva@mail.ru
* Correspondence: aidar@iopc.ru (A.T.G.); yufzuev@mail.ru (Y.F.Z.)
† These authors contributed equally to this work.

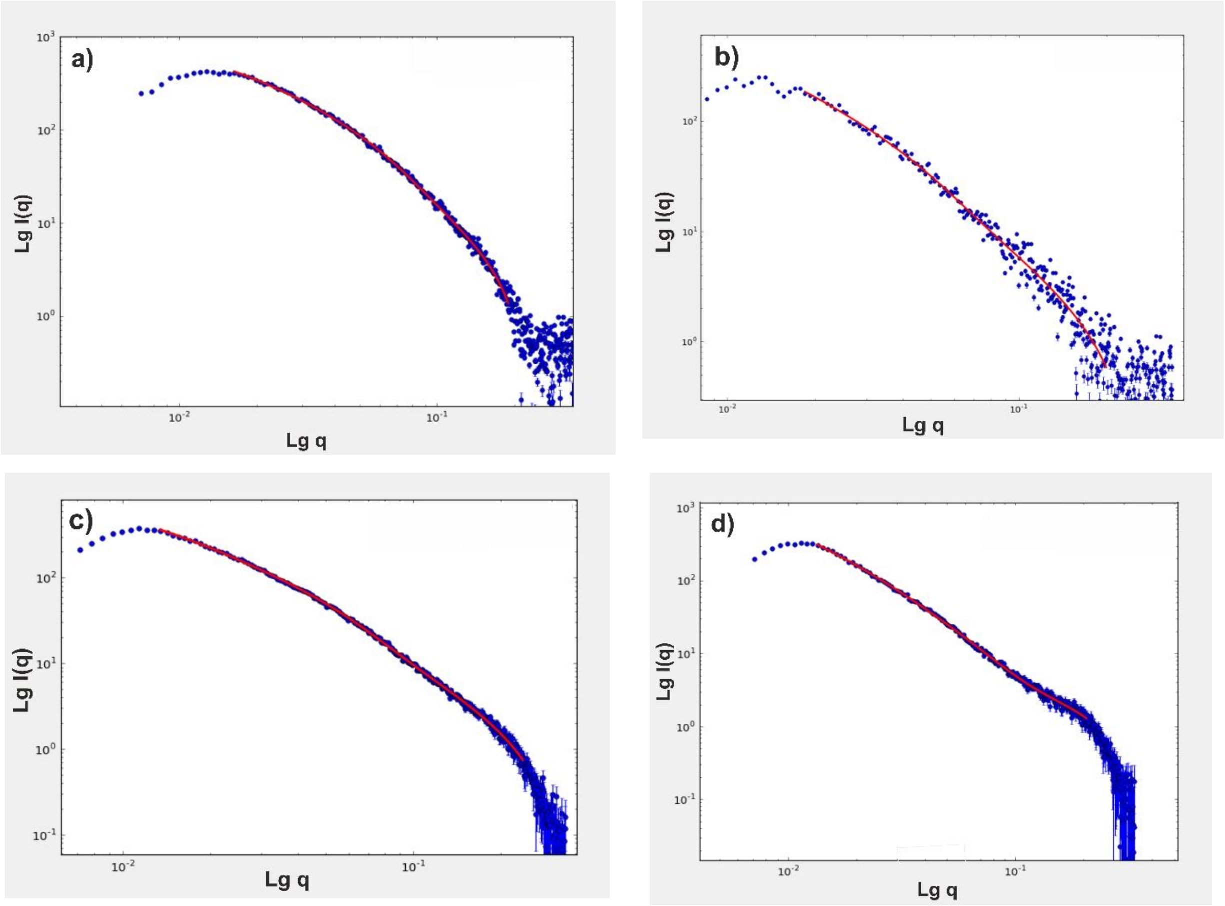

Figure S1. Fitting of experimental SAXS curves *(in logarithmic scale, Lg(I) vs q)* in the Gauss-Lorentz gel model framework for pure hydrogel (a - 14 °C; b - 35 °C) and hydrogel modified by CNT (c - 14 °C; d - 35 °C) after background substruction, experimental points – circles, solid line – calculated curves. Scattering vector q=4πSinθ/λ, $Å^{-1}$.

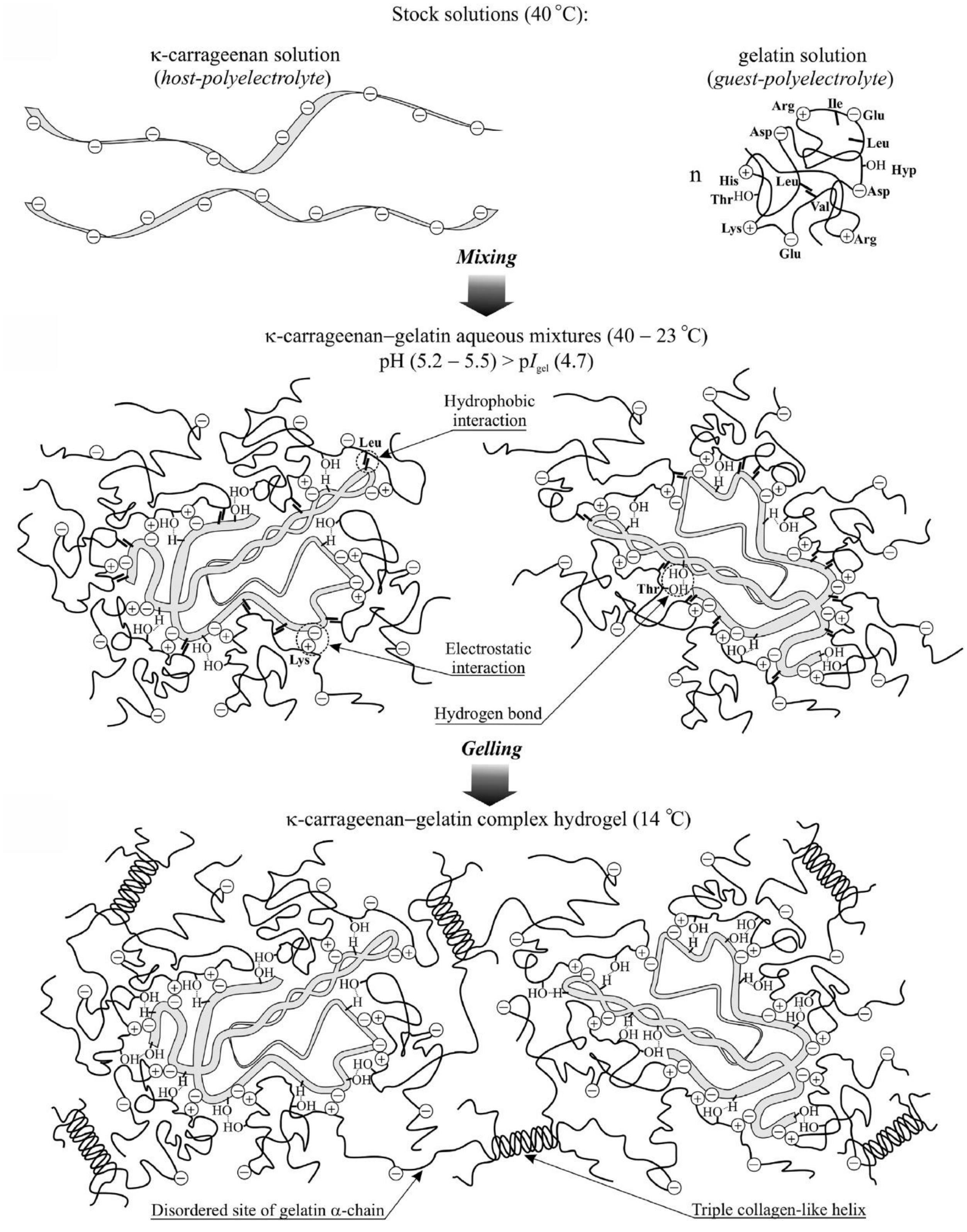

Figure S2. Qualitative scheme of κ-carrageenan-gelatin polyelectrolyte complex and its gelling via triple collagen-like helixes and disordered α-gelatin chains bonding [60].
